# Comparative cytogenetics of tree frogs of the *Dendropsophus marmoratus* (Laurenti, 1768) group: conserved karyotypes and interstitial telomeric sequences

**DOI:** 10.3897/CompCytogen.v10i4.9972

**Published:** 2016-12-14

**Authors:** Lívia S. R. Teixeira, Karin Regina Seger, Cíntia Pelegrineti Targueta, Victor G. Dill Orrico, Luciana Bolsoni Lourenço

**Affiliations:** 1 Departamento de Biologia Estrutural e Funcional, Instituto de Biologia, Universidade Estadual de Campinas, 13083-863 Campinas, São Paulo, Brasil; 2 Departamento de Ciências Biológicas, Universidade Estadual de Santa Cruz, 45662-900 Ilhéus, Bahia, Brasil

**Keywords:** Chromosomes, Anura, telomeric sequence

## Abstract

The diploid number 2n = 30 is a presumed synapomorphy of *Dendropsophus* Fitzinger, 1843, although a noticeable variation in the number of biarmed/telocentric chromosomes is observed in this genus. Such a variation suggests that several chromosomal rearrangements took place after the evolutionary origin of the hypothetical ancestral 30-chromosome karyotype; however, the inferred rearrangements remain unknown. Distinct numbers of telocentric chromosomes are found in the two most cytogenetically studied species groups of *Dendropsophus*. In contrast, all three species of the *Dendropsophus marmoratus* (Laurenti, 1768) group that are already karyotyped presented five pairs of telocentric chromosomes. In this study, we analyzed cytogenetically three additional species of this group to investigate if the number of telocentric chromosomes in this group is not as variable as in other *Dendropsophus* groups. We described the karyotypes of *Dendropsophus seniculus* (Cope, 1868), *Dendropsophus soaresi* (Caramaschi & Jim, 1983) and *Dendropsophus novaisi* (Bokermann, 1968) based on Giemsa staining, C-banding, silver impregnation and *in situ* hybridization with telomeric probes. *Dendropsophus seniculus*, *Dendropsophus soaresi* and *Dendropsophus novaisi* presented five pairs of telocentric chromosomes, as did the remaining species of the group previously karyotyped. Though the species of this group show a high degree of karyotypic similarity, *Dendropsophus soaresi* was unique in presenting large blocks of het-ITSs (heterochromatic internal telomeric sequences) in the majority of the centromeres. Although the ITSs have been interpreted as evidence of ancestral chromosomal fusions and inversions, the het-ITSs detected in the karyotype of *Dendropsophus soaresi* could not be explained as direct remnants of ancestral chromosomal rearrangements because no evidence of chromosomal changes emerged from the comparison of the karyotypes of all of the species of the *Dendropsophus marmoratus* group.

## Introduction

Faivovich et al. (2005) resurrected the genus *Dendropsophus* Fitzinger, 1843 to accommodate all Neotropical hylid species known or suspected to have a diploid chromosome number 2n = 30. This cytogenetic character state was later confirmed as a synapomorphy for this genus by Suárez et al. (2013) after the description of a 2n = 24 karyotype for *Xenohyla* Izecksohn, 1998, the sister genus of *Dendropsophus* (see Faivovich et al. 2005, Pyron and Wiens 2011, Duellman et al. 2016). Based on preliminary data, Bogart (1973) hypothesized that centric fission events may have been involved in the origin of an ancestral 30-chromosome karyotype, which is a hypothesis that was also considered by Suárez et al. (2013). However, the chromosomes that are putatively involved in these events have not yet been recognized, and this hypothesis remains to be validated.

Although all of the *Dendropsophus* species karyotyped to date show 2n = 30 (see review in Catroli and Kasahara 2009, Medeiros et al. 2013, Suárez et al. 2013, Oliveira et al. 2016), a noticeable variation in the number of biarmed/telocentric chromosomes is observed among them, suggesting that several chromosomal rearrangements took place after the evolutionary origin of the hypothetical ancestral 30-chromosome karyotype. Karyotypes with only biarmed chromosomes [as in *Dendropsophus minutus* (Peters, 1872) (Gruber et al. 2005) and *Dendropsophus leali* (Bokermann, 1964) (Bogart 1973)] and karyotypes with up to five pairs of telocentric/subtelocentric chromosomes [as in *Dendropsophus labialis* (Peters, 1863) (Bogart 1973), *Dendropsophus sanborni* (Schmidt, 1944) and *Dendropsophus jimi* (Napoli & Caramaschi, 1999) (Medeiros et al. 2013)] may be observed. However, the chromosomes and events involved in these rearrangements also remain undiscovered because most *Dendropsophus* species karyotypes are not yet described, and few chromosomal markers are available for the known karyotypes, preventing reliable hypotheses of chromosome homeology.

Of the nine species groups recognized in *Dendropsophus* (for a review of the *Dendropsophus* groups, see Faivovich et al. 2005), the *Dendropsophus microcephalus* (Cope, 1886) group is the most species-rich (currently with 40 species—Frost 2016) and the most studied cytogenetically (17 species karyotyped—review by Catroli and Kasahara 2009, Medeiros et al. 2013, Oliveira et al. 2016). It is noteworthy that karyotypes without any telocentric chromosome (in *Dendropsophus leali*—Bogart 1973) and with one [as in *Dendropsophus bipunctatus* (Spix, 1824)—Bogart 1973], two [as in *Dendropsophus phlebodes* (Steineger, 1906)—Kaiser et al. 1996], three [as in *Dendropsophus cruzi* (Pombal & Bastos, 1998)—Gruber et al. 2005], four [as in *Dendropsophus nanus* (Boulenger, 1889)—Medeiros et al. 2003] or five (as in *Dendropsophus jimi*—Medeiros et al. 2013) telocentric chromosome pairs are observed in this group. Karyotypes with distinct numbers of telocentric chromosomes were also found in the *Dendropsophus leucophyllatus* (Beireis, 1783) group (Bogart 1973, Kaiser et al. 1996, Gruber et al. 2005), which currently has 11 species (see Frost 2016) and is the second most cytogenetically studied species group of *Dendropsophus* (four of the named species are karyotyped). In contrast, all of the three species of the *Dendropsophus marmoratus* group that are already karyotyped (i.e. *Dendropsophus marmoratus*, *Dendropsophus melanargyreus* and *Dendropsophus nahdereri*) present five pairs of telocentric chromosomes (Bogart 1973, Gruber et al. 2005, Suárez et al. 2013).

Chromosomal sites composed of telomeric repeats localized apart from the telomeres, also known as interstitial or intrachromosomal telomeric sequences (ITSs) or repeats (ITRs), have been detected in several animals (Meyne et al. 1990, Nanda et al. 2002, Rovatsos et al. 2015, Schmid and Steinlein 2016) and plants (Tek and Jiang 2004, He et al. 2013). Based on the genomic location and sequence organization, especially in the number of telomeric repeats, Ruiz-Herrera et al. (2008) classified the ITSs in short ITSs (s-ITSs) and heterochromatic ITS (het-ITS). The s-ITSs [called short interstitial telomeres, short ITs, by Azzalin at al. (2001)] are short stretches of telomeric hexamers distributed at internal chromosomal positions, presumably present in all vertebrate species, whereas het-ITSs are large blocks of telomeric-like repeats localized mainly in centromeric and pericentromeric regions (Ruiz-Herrera et al. 2008). The s-ITSs probably originated from the insertion of telomeric repeats during the repair of DNA double-strand breaks, as was originally proposed by Nergadze et al. (2004, 2007). On the other hand, the het-ITSs have been widely considered to be remnants of ancestral chromosomal rearrangements as fusions (e.g., Lee et al. 1993, Slijepcevic 1998, Ropiquet et al. 2010, Paço et al. 2013, Young et al. 2013) and inversions (e.g., Farré et al. 2009, Ocalewicz et al. 2013, Paço et al. 2013). Recently, Schmid and Steinlein (2016) proposed an additional category of ITS, named euchromatic-ITSs (eu-ITSs), to accommodate the large ITSs that are not revealed as heterochromatic sites by C-banding or staining with base-specific fluorochromes.

The *Dendropsophus marmoratus* group currently includes eight species, i.e., *Dendropsophus marmoratus*, *Dendropsophus acreanus* (Bokermann, 1964), *Dendropsophus dutrai* (Gomes & Peixoto, 1996), *Dendropsophus melanargyreus*, *Dendropsophus nahdereri*, *Dendropsophus novaisi*, *Dendropsophus seniculus* and *Dendropsophus soaresi* (Faivovich et al. 2005). Some adult and larval morphological synapomorphies of this species group may be recognized (Faivovich et al. 2005); however, its cladistic proximity with other *Dendropsophus* species groups as well as the internal phylogenetic relationships of this group remain unclear (Faivovich et al. 2005; Pyron and Wiens 2011, Fouquet et al. 2011, Medeiros et al. 2013). To date, up to three of the eight species of the *Dendropsophus marmoratus* group have been included in phylogenetic analysis (Fouquet et al. 2011).

In this study, we analyzed cytogenetically three additional species of the *Dendropsophus marmoratus* group to investigate if the number of telocentric chromosomes in this group is not as variable as in other *Dendropsophus* groups. Because karyotypic variation in number of telocentric chromosomes may result from rearrangements involving telomeric sequences (review in Ruiz-Herrera et al. 2008), we included here the mapping of telomeric sequences in the karyotypes of two of the analyzed species. Additionally, we provided the nucleotide sequence of a fragment of the 16S ribosomal RNA gene of one exemplar for each of the species that were analyzed cytogenetically to yield a reliable association of the chromosomal data set with a DNA data set that has been remarkably useful for taxonomic and phylogenetic studies of anurans.

## Material and methods

### Specimens

Four male exemplars of *Dendropsophus seniculus* from Ribeirão Grande, state of São Paulo, Brazil, nine *Dendropsophus soaresi* males from Barreiras, state of Bahia, Brazil and one female of *Dendropsophus novaisi* from Jequié, state of Bahia, Brazil were analyzed cytogenetically. The specimens were collected under a permit issued by the Instituto Brasileiro do Meio Ambiente e dos Recursos Naturais Renováveis (IBAMA) (#32483), and deposited at the amphibian collection of the Museu de Zoologia “Prof. Adão José Cardoso” at the Institute of Biology – University of Campinas, Campinas, Brazil, under the accession numbers ZUEC 17225–17228 (*Dendropsophus seniculus*), ZUEC 16867–16875 (*Dendropsophus soaresi*) and ZUEC 17858 (*Dendropsophus novaisi*).

### Cytogenetic analyses

Animals were injected intraperitoneally with 2% colchicine (Sigma – Aldrich; 0.02 mL per 1 g body weight) for an “in vivo” treatment that lasted at least 4 hours. The animals were deeply anesthetized with lidocaine gel 2% and their intestines were removed and used for obtaining chromosomal preparations according to the method of King and Rofe (1976). Chromosomes were conventionally stained with 10% Giemsa and sequentially submitted to C-banding (Sumner 1972) and silver staining by the Ag-NOR method (Howell and Black 1980).

To localize telomeric sequences, the karyotypes were *in situ* hybridized with the probe (CCCTAA)_3_ (PNA – Peptid Nucleic Acid TelC-Cy3; PNA Bio Inc.), following the manufacturer’s instructions.

### Mitochondrial DNA sequences

Samples of genomic DNA were obtained from *Dendropsophus seniculus* (ZUEC 17225), *Dendropsophus soaresi* (ZUEC 16867) and *Dendropsophus novaisi* (ZUEC 17858) following the procedure reported by Medeiros et al. (2013). A fragment of approximately 1300 bp of the 16S ribosomal RNA gene was PCR-amplified using the primers 12L13(L) (Feller and Hedges 1998) and 16Sbr(H) (Palumbi et al. 1991). The amplified products were purified with the GFX PCR and Gel Band DNA purification Kit (GE Healthcare) and directly sequenced in an automatic DNA ABI/Prism sequencer (Applied Biosystems) using BigDye Terminator kits (Applied Biosystems) and the primers 12L13 (Feller and Hedges 1998), TitusI(H) (Titus 1992), Hedges16L2a (Hedges 1994), Hedges16H10 (Hedges 1994), 16Sar(L) (Palumbi et al. 1991) and 16Sbr(H) (Palumbi et al. 1991). DNA sequences were aligned using ClustalW option implemented in BioEdit v. 7.2.5 (Hall 1999) and compared to each other and to the 16S rDNA sequence of *Dendropsophus seniculus* available at GenBank (AY843666).

## Results

### Cytogenetic analyses

**Figure 1.**
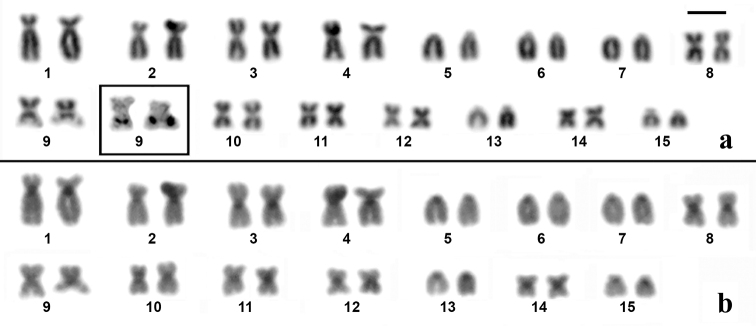
Karyotype of *Dendropsophus seniculus* stained with Giemsa (**a**) and C-banded (**b**). In the inset in (a), the NOR-bearing chromosome pair 9 after silver staining. Bar = 5 µm.

**Figure 2.**
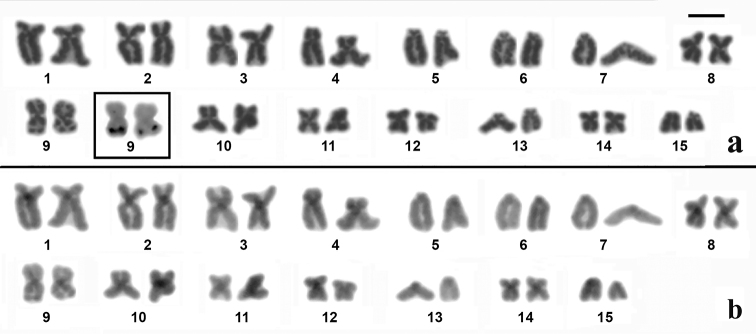
Karyotype of *Dendropsophus soaresi* stained with Giemsa (**a**) and C-banded (**b**). In the inset in (**a**), the NOR-bearing chromosome pair 9 after silver staining. Bar = 5 µm.

**Figure 3.**
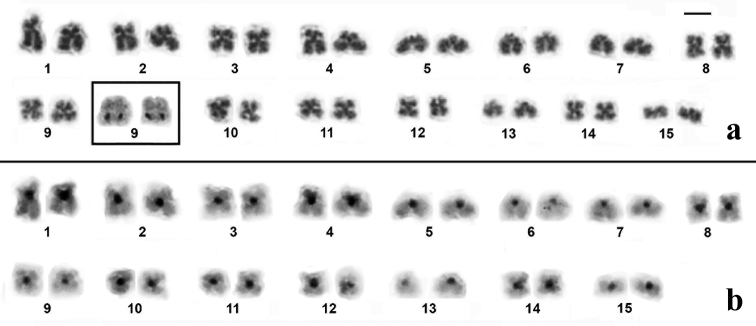
Karyotype of *Dendropsophus novaisi* stained with Giemsa (**a**) and C-banded (**b**). In the inset in (**a**), the NOR-bearing chromosome pair 9 after silver staining. Bar = 5 µm.

The karyotypes of *Dendropsophus seniculus*, *Dendropsophus soaresi* and *Dendropsophus novaisi* were very similar and presented three pairs (pairs 1, 2 and 4) of submetacentric chromosomes, seven pairs (pairs 3, 8–12 and 14) of metacentric chromosomes and five pairs (pairs 5–7, 13 and 15) of telocentric chromosomes (Figures 1–3). The nucleolus organizer region (NOR) was detected by silver staining in the long arm of chromosome 9 of the three species (insets in Figures 1–3). C-banding only detected the centromeric region of all the chromosomes of *Dendropsophus seniculus* (Figure 1b), *Dendropsophus soaresi* (Figure 2b) and *Dendropsophus novaisi* (Figure 3b).

**Figure 4.**
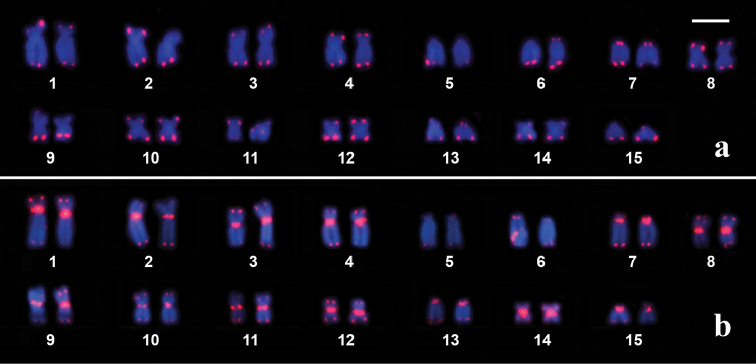
Karyotypes of *Dendropsophus seniculus* (**a**) and *Dendropsophus soaresi* (**b**) hybridized with telomeric probe.

*In situ* hybridization detected telomeric sequences in all of the telomeres of *Dendropsophus seniculus* and *Dendropsophus soaresi* (Figure 4). Additionally, interstitial telomeric sequences (ITSs) were detected in the centromeres of the chromosomes of *Dendropsophus soaresi*, except in two of the five pairs of telocentric chromosomes (pairs 5 and 6) (Figure 4b).

### Mitochondrial DNA sequences

The nucleotide sequence (1312 bp) (see Suppl. material 1) of the 16S rDNA of the specimen of *Dendropsophus seniculus* that we analyzed was highly similar (99.6%) to the corresponding sequence available at GenBank (AY843666; Faivovich et al. 2005) from a specimen of *Dendropsophus seniculus* from Angra dos Reis, Rio de Janeiro State, Brazil. The sequences obtained from *Dendropsophus soaresi* (1314 bp) and *Dendropsophus novaisi* (1310 bp) (see Suppl. material 1) were 86.4% similar to each other, and 88.4% and 90.7% (average value) similar to the sequences of *Dendropsophus seniculus*, respectively.

## Discussion

### Karyotypic comparisons

The three species analyzed showed karyotypes composed of five pairs of telocentric chromosomes, similarly to the other three species of the *Dendropsophus marmoratus* group previously studied cytogenetically [i.e. *Dendropsophus marmoratus* (Bogart 1973, Suárez et al. 2013), *Dendropsophus melanargyreus* (Suárez et al. 2013) and *Dendropsophus nahdereri* (Gruber et al. 2005)]. The interspecific morphological conservation of the karyotypes of the species of the *Dendropsophus marmoratus* group contrasts with the variation found in other *Dendropsophus* groups. The *Dendropsophus microcephalus* group, for instance, includes species with zero to five pairs of telocentric chromosomes (see Bogart 1973, Kaiser et al. 1996, Gruber et al. 2005, Medeiros et al. 2003). Variation in the number of telocentric chromosomes could also be found in the *Dendropsophus leucophyllatus* group, although only four named species of this group have been karyotyped (see Bogart 1973, Kaiser et al. 1996, Gruber et al. 2005).

According to the estimated dates of divergence provided by Duellman et al. (2016), the *Dendropsophus marmoratus*, *Dendropsophus microcephalus* and *Dendropsophus leucophyllatus* groups arose at similar times in the mid-Miocene (17.0, 17.2 and 18.7 Mya, respectively). Therefore, differential time for divergence does not justify the different levels of karyotypic variation observed among the three aforementioned species groups. Further analyses of the chromosomal rearrangements involved in the karyotypic variations in *Dendropsophus* combined with phylogeographic studies are still necessary to elucidate about the high conservation in the number of telocentric chromosomes in the *Dendropsophus marmoratus* group.

With respect to the number and relative size of the telocentric chromosomes, the karyotypes of the species of the *Dendropsophus marmoratus* group are similar to that of *Dendropsophus labialis* (Bogart 1973), a species included in the *Dendropsophus labialis* group. This morphological similarity suggests that the telocentric chromosomes of these karyotypes could be homeologous, although a better characterization of these chromosomes is fundamental to test this hypothesis. According to the most comprehensive phylogenetic analysis of *Dendropsophus* (Duellman et al. 2016) and assuming the telocentric chromosomes of the species of the *Dendropsophus marmoratus* group and *Dendropsophus labialis* are homeologous, it is possible to hypothesize that this karyotype configuration is plesiomorphic with respect to those constituted by other numbers and/or relative sizes of the telocentric chromosomes. However, internal relationships within *Dendropsophus* are consistently poorly supported and small taxonomic additions cause huge impacts (e.g., Fouquet et al. 2015, Duellman et al. 2016).

Five pairs of telocentric chromosomes were also observed in the karyotypes of *Dendropsophus jimi* and *Dendropsophus sanborni* (Medeiros et al. 2013), which are species that belong to the *Dendropsophus microcephalus* group. In these karyotypes, however, the telocentric pairs were classified as pairs 5, 6, 12, 13 and 15, whereas the telocentric chromosomes of the karyotypes of *Dendropsophus labialis* and the species of the *Dendropsophus marmoratus* group are numbered as pairs 5, 6, 7, 13 and 15. Because only a few chromosomal markers are available for a comparison of these karyotypes, it is still not possible to determine if the telocentric chromosomes of all of these species are homeologous. Therefore, we cannot discard the possibility that chromosomes 12 of *Dendropsophus jimi* and *Dendropsophus sanborni* are homeologous to chromosomes 7 of *Dendropsophus labilais* and the species of the *Dendropsophus marmoratus* group, although these chromosomes differ by the presence of NOR in chromosomes 12 of *Dendropsophus jimi* and *Dendropsophus sanborni* (Medeiros et al. 2013).

The similarities among the karyotypes of the species of the *Dendropsophus marmoratus* group are not restricted to the number of telocentric chromosomes. *Dendropsophus seniculus*, *Dendropsophus soaresi* and *Dendropsophus novaisi* also share with *Dendropsophus marmoratus* and *Dendropsophus melanargyreus* the location of the NOR at a distal site of the long arm of chromosome 9, which differs from *Dendropsophus nahdereri*, whose NOR is located on the short arm of the submetacentric chromosome 1 (Gruber et al. 2005).

C-banding did not reveal any differential band that could be considered exclusive to the karyotypes of *Dendropsophus seniculus*, *Dendropsophus soaresi* or *Dendropsophus novaisi*, since only the centromeric regions were detected by this technique (present work). Conspicuous non-centromeric C-bands were also absent in the karyotypes of *Dendropsophus marmoratus* and *Dendropsophus melanargyreus*, the other two species of the *Dendropsophus marmoratus* group whose karyotypes were already C-banded, although Suárez et al. (2013) reported the presence of some distal and interstitial C-bands in those karyotypes.

Despite the high similarity of the karyotypes of the species of the *Dendropsophus marmoratus* group with respect to the number and morphology of the chromosomes, C-banding pattern and location of NOR (except for *Dendropsophus nahdereri*), the karyotype of *Dendropsophus soaresi* stands out because of the presence of internal telomeric sequences in addition to the terminal telomeric sequences.

### Interstitial telomeric sequences

Large and short ITSs are likely to play a role in karyotypic evolution. Several studies support the hypothesis that, in addition to possibly representing relics of chromosomal changes, the het-ITSs may themselves induce chromosome breakage and subsequent chromosomal rearrangements (reviewed in Ruiz-Herrera et al. 2008 and Bolzán 2012). Similarly, experimental and associative studies have also suggested the involvement of s-ITSs with genomic instability or chromosomal hot spots of recombination (Aksenova et al. 2013, Wood et al. 2015).

The het-ITSs detected in the present study in the karyotype of *Dendropsophus soaresi* cannot be explained as direct remnants of ancestral chromosomal rearrangements because no evidence of chromosomal changes has emerged from the comparison of the karyotypes of all species of the *Dendropsophus marmoratus* group already known (Bogart 1973, Gruber et al. 2005, Suárez et al. 2013, present work). Although it is very similar to the karyotypes of the other species of the group, the karyotype of *Dendropsophus soaresi* is unique in presenting large blocks of centromeric ITSs because the karyotypes of *Dendropsophus seniculus* (present study), *Dendropsophus melanargyreus* and *Dendropsophus marmoratus* (Suárez et al. 2013) showed only telomeric sites hybridized with telomeric probes.

The occurrence of het-ITSs at the majority of the centromeres of the karyotype of *Dendropsophus soaresi* is also remarkable and suggests the expansion and homogenization of telomeric sequences throughout the repetitive elements that compose these centromeric regions. Repetitive DNA, such as centromeric satellite DNA, is expected to expand in the genome and evolve in concert by a series of mechanisms, including unequal crossing-over, gene conversion, rolling circle replication and reinsertion, and transposon-mediated exchange (see Dover 1982, 1986 and the review by Plohl et al. 2008). The telomeric repeats present in heterochromatic sites should be subject to the same evolutionary forces (see Ruiz-Herrera et al. 2008). In contrast, the absence of het-ITS in the centromere of two chromosome pairs (telocentric chromosome pairs 5 and 6) of *Dendropsophus soaresi* suggests that these centromeres do not evolve in concert with the remaining centromeric regions of the genome. The reason for such differential behavior is intriguing and remains unknown.

Similar to observations of *Dendropsophus soaresi*, large blocks of centromeric/pericentromeric ITSs that were widely distributed throughout the genome were previously found in four other hylid species [i.e. *Aplastodiscus albofrenatus* (Lutz, 1924), *Aplastodiscus arildae* (Cruz & Peixoto, 1987) and *Aplastodiscus eugenioi* (Carvalho-e-Silva & Carvalho-e-Silva, 2005)—Carvalho et al. 2009, Gruber et al. 2012a; *Hypsiboas faber* (Wied-Neuwied, 1821)—Schmid and Steinlein 2016]. In the hylid *Itapotihyla langsdorffii* (Duméril & Bibron, 1841), ITSs were also observed in several centromeres (Gruber et al. 2012b), but in this case the het-ITSs are not as large as those previously mentioned. In addition to the aforementioned hylids, other fifteen hylid species showed ITSs in their karyotypes (Meyne et al. 1990, Wiley et al. 1992, Suárez et al. 2013, Mattos et al. 2014, Bruschi et al. 2014, Schmid and Steinlein 2016), which suggests that the appearance of this type of sequence is recurrent in the Hylidae family. Only the centromeric ITS found in chromosome 3 of *Scarthyla goinorum* (Bokermann, 1962) was clearly interpreted as a remnant of a chromosomal fusion that in that case could respond to the reduced chromosome number observed in this species (Suárez et al. 2013). The insertion of telomeric repeats during the repair of double-strand breaks in DNA as a phenomenon putatively involved in the origin of ITS in Hylidae remains unexplored.

It is worth noticing that in the sample of metaphases analyzed in this paper, large signals of the telomeric probe were detected at a subterminal non-heterochromatic site of some chromosomes of *Dendropsophus seniculus* (Figure 4a, at the long arm of the right homologous of chromosome 9). This hybridization pattern resembles that pattern interpreted by Wood et al. (2014, 2015) as cytological evidence of the occurrence of t-loops formed between telomere and s-ITS. However, studies designed to search for s-ITSs in hylid karyotypes have not yet been performed, and the prevalence of ITSs in Hylidae remains an intriguing question to be assessed in further studies.

### Association between cytogenetic data and 16S rDNA sequences

The high similarity between the 16S rDNA sequence of *Dendropsophus seniculus* we provided and that previously obtained by Faivovich et al. (2005) enables a reliable association between the cytogenetic data shown here and the analyses hitherto conducted with the previously available sequence, including the studies of Fouquet et al. (2015) and Duellman et al. (2016). On the other hand, the nucleotide sequences obtained here from *Dendropsophus soaresi* and *Dendropsophus novaisi* were the first report of 16S rDNA sequences for these species.

*Dendropsophus* systematics are in flux and even comprehensive datasets are unable to provide a stable historical hypothesis (Fouquet et al. 2011; Peloso et al. 2016). The association between the cytogenetic dataset and 16S rDNA sequences may be very helpful in future analyses, especially because the species-level taxonomy of *Dendropsophus* has been subject to several changes. A number of *Dendropsophus* species has been described in the last few years (Rivera-Correia and Orrico 2013, Ortega-Andrade and Ron 2013, Orrico et al. 2014, Fouquet et al. 2015, Peloso et al. 2016) as well as species synonymizaton has been proposed (Guarnizo et al. 2012, Orrico et al. 2013). Therefore, a reliable association between different sets of data is fundamental for further integrative studies.

## Conclusion

All of the karyotypes found in the *Dendropsophus marmoratus* group to date showed five pairs of telocentric chromosomes and were also similar in the location of NORs (except for the *Dendropsophus nahdereri* karyotype, described by Gruber et al. 2005) and C-banding pattern. Because of this karyotypic conservatism, the het-ITSs present in the majority of the centromeres of the karyotype of *Dendropsophus soaresi* may not be interpreted as direct remnants of ancestral chromosomal rearrangements.

## Supplementary materials

Supplementary material 1 16S rDNA sequences Data type: Tif file Explanation note: Alignment of 16S rDNA fragments obtained from specimens of *Dendropsophus seniculus*, *Dendropsophus novaisi* and *Dendropsophus soaresi* that were used in cytogenetic analyses. GenBank accession numbers: KY053469 (*Dendropsophus seniculus*), KY053470 (*Dendropsophus novaisi*), and KY053471 (*Dendropsophus soaresi*). This dataset is made available under the Open Database License (http://opendatacommons.org/licenses/odbl/1.0/). The Open Database License (ODbL) is a license agreement intended to allow users to freely share, modify, and use this Dataset while maintaining this same freedom for others, provided that the original source and author(s) are credited.Lívia S. R. Teixeira, Karin Regina Seger, Cíntia Pelegrineti Targueta, Victor G. Dill Orrico, Luciana Bolsoni Lourenço
