# Supplementary figures and images for: Comparative cytogenetics of tree frogs of the Dendropsophus marmoratus (Laurenti, 1768) group: conserved karyotypes and interstitial telomeric sequences
Source: Comp Cytogenet. 2016 Dec 14;10(4):753–67. doi: 10.3897/CompCytogen.v10i4.9972 (PMC5240522; doi:10.3897/CompCytogen.v10i4.9972)

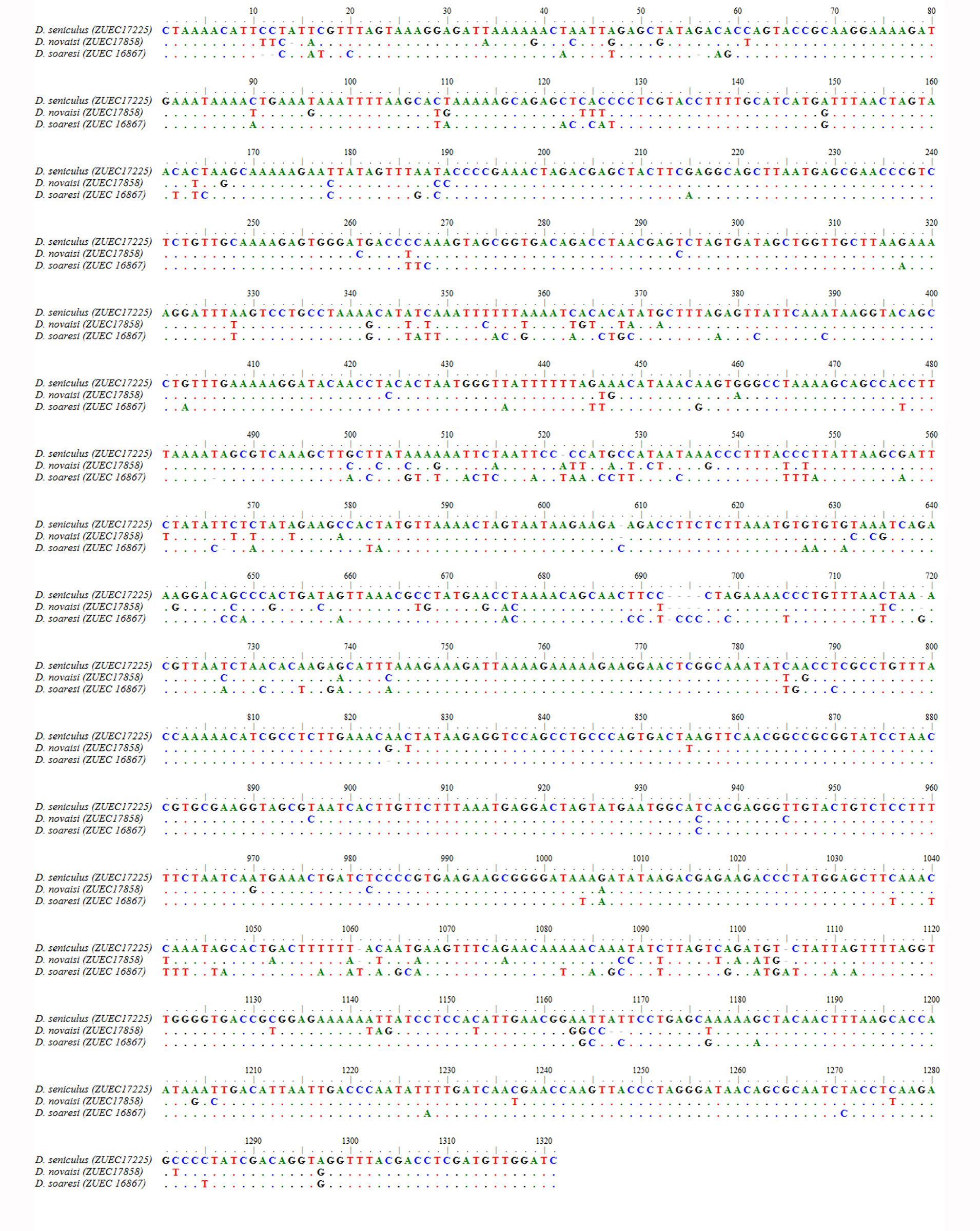

Supplement: Supplementary material 1 — 16S rDNA sequences [file CompCytogen-010-753-s001.tif]
